# The complete mitochondrial genome sequence of the intertidal crab *Parasesarma Tripectinis* (Arthropoda, Decapoda, Sesarmidae)

**DOI:** 10.1080/23802359.2018.1437804

**Published:** 2018-02-10

**Authors:** Yeong-Jun Park, Chang Eon Park, Seok Hyun Lee, Hyun Sook Ko, Ihsan Ullah, Ui Wook Hwang, Jae-Ho Shin

**Affiliations:** a School of Applied Biosciences, Kyungpook National University, Daegu, Republic of Korea;; b Department of Life Science, Silla University, Busan, Republic of Korea;; c Department of Biological Sciences Faculty of Science, King Abdulaziz University, Jeddah, Saudi Arabia;; d Department of Biology, Teachers College & Institute for Phylogenomics and Evolution Kyungpook National University, Daegu, Republic of Korea

**Keywords:** *Parasesarma tripectinis*, intertidal crab, Sesarmidae

## Abstract

*Parasesarma tripectinis* is known as an intertidal crab and inhabits Asian region. This crab has larval release at semilunar rhythm. Here, we report the complete sequence of the mitochondrial genome (mitogenome), which is composed of 15,612 base pair (bp) encoding 13 protein-coding genes, 22 transfer RNAs, 2 ribosomal RNAs, and an A + T rich region. The nucleotide composition of *P. tripectinis* was G + C: 25.8%, A + T: 74.2%, with a strong AT bias. Phylogenetic analysis using whole mitogenome figured out that *P. tripectinis* was closely related to *Sesarma neglectum* which belongs to the same family Sesarmidae.

*Parasesarma tripectinis* is classified in the family Sesarmidae, and it inhabits intertidal area (Dai et al. 1991; Ng et al. 2008). This intertidal crab was first discovered in 1940 by a Chinese zoologist, Shen Chia-Jui (Shen 1940). This species spreads throughout at intertidal coastal zones in Asia and exhibits semilunar rhythm in reproductive activity by releasing larva (Saigusa 1981; Hsueh 2002). In general, specific genes such as phosphoenolpyruvate carboxykinase (PEPCK), sodium-potassium ATPase α-subunit (NaK), 16S rRNA (LSU), and cytochrome oxidase subunit I (*cox*1) are compared for phylogenetic classification of crabs (Schubart et al. 2001; Tsang et al. 2008). However, it is considered to be more certain to compare whole mitogenomes if possible.

A sample of *P. tripectinis* was collected in Gangseo-gu, Busan, Republic of Korea (GIS: 35°04’47”N 128°52'36”E). The sample was kept in National Institute of Biological Resources in Korea under the voucher number NIBRGR0000072156. Mitogenomic DNA of *P. tripectinis* was extracted from muscle tissue sample using QIAamp Tissue Kit (Qiagen, Valencia, CA) (Graham 2001). The amplification of mitogenome was performed using a set of mitochondrial specific primers (Martin et al. 2016). In PCR cycle, lower extension temperature was used for A + T rich region amplification (Su et al. 1996). Next generation sequencing was carried out using the Ion Torrent PGM machine (Life Technologies/Thermo Fisher Scientific, Waltham, MA). Whole mitogenome assembly and annotation were performed using CLC genomics workbench 8.5 (CLC Bio, Denmark). Protein coding genes (PCGs), ribosomal RNA genes (rRNAs), transfer RNA genes (tRNAs) and D-loop were confirmed using NCBI Basic Local Alignment Search Tool (BLAST) (Altschul et al. 1990) and tRNA-scan 1.21 (Lowe and Eddy 1997), respectively.

The complete mitogenome of *P. tripectinis* was composed of circular DNA with 15,612 bp containing 13 PCGs, 22 tRNAs, 2 rRNAs and a D-loop. The mitogenome sequence was submitted to GenBank under accession number NC030046. Thirteen PCGs use ATG, ATT or ATA as a start codon. Incomplete stop codons in *cox1*, *cox2*, *cox3*, and *cytb* were found as T(aa) and TA(a) (Yamauchi et al. 2003; Hou et al. 2007; Zhang et al. 2016). The nucleotide composition was asymmetric (A: 36.2%, T: 38.0%, G: 10.1%, C: 15.7%) with a strong AT bias. Phylogenetic analysis was done with 15 mitogenomes in Decapoda order. Maximum likelihood phylogenetic tree was obtained using MEGA 6.0 (Tamura et al. 2013) with bootstrap methods (1000 replicates). The resultant phylogenetic tree indicated that *P. tripectinis* was mostly closed with *Sesarma neglectum* (NC031851) which is located in same family and super family (Figure 1). Based on these results, mitogenome of *P. tripectinis* could contribute to the phylogenetic knowledge of the family Sesarmidae.

**Figure 1.**
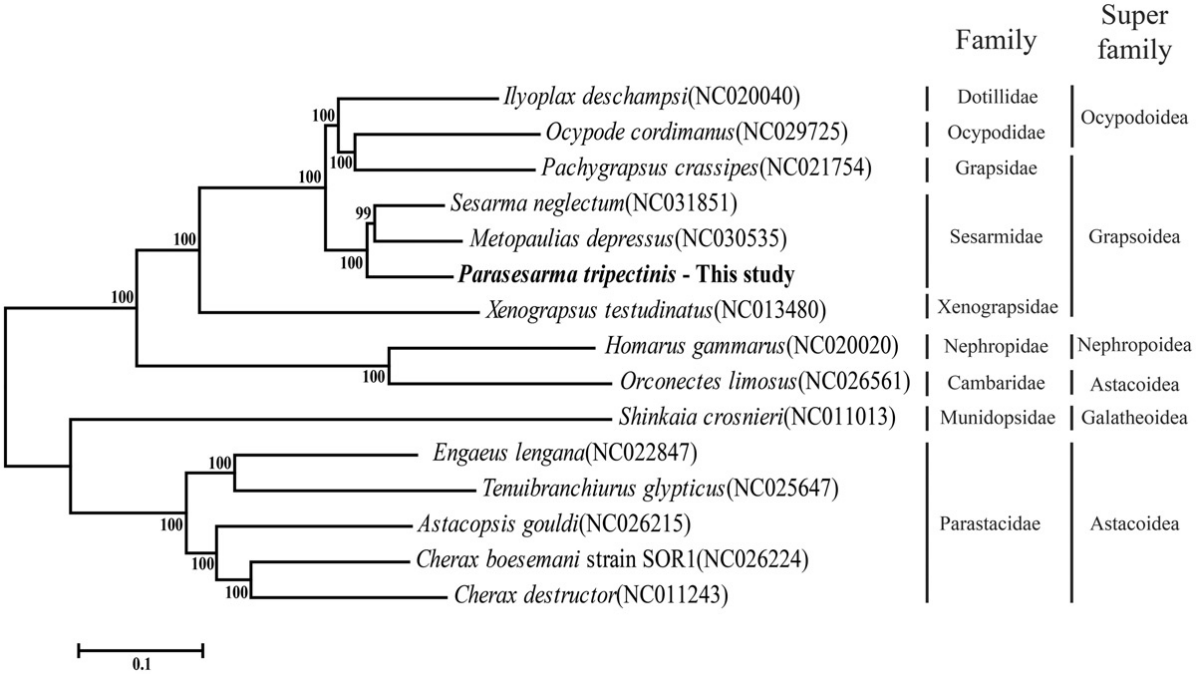
Phylogenetic tree based on 15 whole mitogenomes constructed using maximum likelihood approach. The number in phylogenetic tree is bootstrap probability value. On right side, vertical stick indicated specific family and super family of crab in Decapoda order. The GenBank accession numbers are indicated after the scientific name.
